# Does higher performance in a national licensing examination predict better quality of care? A longitudinal observational study of Ethiopian anesthetists

**DOI:** 10.1186/s12871-024-02575-w

**Published:** 2024-05-27

**Authors:** Yohannes Molla Asemu, Tegbar Yigzaw, Firew Ayalew Desta, Fedde Scheele, Thomas van den Akker

**Affiliations:** 1 Health Workforce Improvement Program, Jhpiego, an affiliate of Johns Hopkins University, Ethiopia country office, Addis Ababa, Ethiopia; 2 Athena Institute, Faculty of Science, Vrije Universiteit Amsterdam, Amsterdam, the Netherlands; 3 Department of Obstetrics and Gynecology, OLVG Teaching Hospital, Amsterdam, the Netherlands; 4 Department of Obstetrics and Gynecology, Amsterdam University Medical Center (AUMC), Amsterdam, the Netherlands; 5 Department of Obstetrics and Gynaecology, Leiden University Medical Centre, Leiden, the Netherlands

**Keywords:** Associate clinicians, Anesthetists, Licensing examination, Quality of care, Patient outcome

## Abstract

**Background:**

Ethiopia made a national licensing examination (NLE) for associate clinician anesthetists a requirement for entry into the practice workforce. However, there is limited empirical evidence on whether the NLE scores of associate clinicians predict the quality of health care they provide in low-income countries. This study aimed to assess the association between anesthetists’ NLE scores and three selected quality of patient care indicators.

**Methods:**

A multicenter longitudinal observational study was conducted between January 8 and February 7, 2023, to collect quality of care (QoC) data on surgical patients attended by anesthetists (*n* = 56) who had taken the Ethiopian anesthetist NLE since 2019. The three QoC indicators were standards for safe anesthesia practice, critical incidents, and patient satisfaction. The medical records of 991 patients were reviewed to determine the standards for safe anesthesia practice and critical incidents. A total of 400 patients responded to the patient satisfaction survey. Multivariable regressions were employed to determine whether the anesthetist NLE score predicted QoC indicators.

**Results:**

The mean percentage of safe anesthesia practice standards met was 69.14%, and the mean satisfaction score was 85.22%. There were 1,120 critical incidents among 911 patients, with three out of five experiencing at least one. After controlling for patient, anesthetist, facility, and clinical care-related confounding variables, the NLE score predicted the occurrence of critical incidents. For every 1% point increase in the total NLE score, the odds of developing one or more critical incidents decreased by 18% (aOR = 0.82; 95% CI = 0.70 = 0.96; *p* = 0.016). No statistically significant associations existed between the other two QoC indicators and NLE scores.

**Conclusion:**

The NLE score had an inverse relationship with the occurrence of critical incidents, supporting the validity of the examination in assessing graduates’ ability to provide safe and effective care. The lack of an association with the other two QoC indicators requires further investigation. Our findings may help improve education quality and the impact of NLEs in Ethiopia and beyond.

**Supplementary Information:**

The online version contains supplementary material available at 10.1186/s12871-024-02575-w.

## Introduction

The Lancet Commission on Global Surgery set minimum targets of 20 surgical specialists (including 4–5 anesthesiologists) per 100,000 populations to perform 5,000 surgical procedures per 100,000 people annually. These targets aim to ensure access to emergency and essential surgery and hasten the journey to universal health coverage (UHC) [1, 2]. However, an estimated five billion people worldwide—up to 95% of the population in sub-Saharan African countries—lack access to basic surgical care, resulting in preventable morbidity and mortality that cost an additional 1.5 million lives each year [3]. The Commission also estimated that at least five million surgical procedures were needed in Ethiopia annually to meet the population’s needs [4]. However, with 0.56 surgical specialists per 100,000 population, no more than 38,000 surgeries were performed in 2012—less than a percent of the estimated need [5].

In response to these unmet surgical needs and World Health Assembly Resolution 68.15 on strengthening surgical care, low- and middle-income countries (LMICs) prioritized surgical access in their national plans and implemented vigorous interventions [6–8]. To achieve the national target of 2,500 procedures per 100,000 people by 2025, the Government of Ethiopia rapidly expanded the surgical workforce, equipped health facilities with essential equipment, and brought surgical service closer to the community by making it available at primary hospitals and health centers levels [9–11]. Along with specialist physician training, the country employed task-sharing and shifting strategies to increase surgical workforce availability by training associate clinician surgical officers and anesthetists. Between 2012 and 2018, Ethiopia increased surgical officers from 43 to 607 and anesthetists from 250 to more than 1,500 [12–14]. Associate clinician anesthetists (also referred to as anesthetists) provide more than 90% of anesthesia care in the country. As a result, 221,260 surgeries (192 per 100,000 population) were performed in 2020/21, an improvement from the 2012 baseline but still far short of national and global targets [10].

However, the task-sharing strategies for surgical care expansion were not without dilemmas. The main concerns include dubious educational quality, subpar graduate competence, poorly defined scope of practice and career pathways, and patient safety issues [13, 15–18]. Besides, while no definitive statement can be made about patient outcomes in the care provided by different groups of the anesthesia workforce [19], studies conducted in Ethiopia revealed a high number of critical incidents attributable to anesthesia care [20, 21]. These concerns indicate the need to strengthen quality assurance and regulatory mechanisms to ensure the quality of patient care and safety.

Ethiopia mandated a national licensing examination (NLE) in 2019 for 13 first-degree graduates, including anesthetists, to ensure that only those new graduates capable of providing safe and effective independent service are licensed to practice healthcare in their respective fields of study [22, 23]. The Ethiopian anesthetist NLE is based on the tasks that anesthetists are required to perform and thus ensures that they are well prepared for on-the-job expectations [17]. The exam is prepared as a single-step cognitive assessment that consists of 180–200 context-based multiple-choice questions designed to assess the application of scientific knowledge across the five competency domains: patient care, professionalism, leadership and management, research, and health promotion and disease prevention. Over 100,000 baccalaureate graduate health professionals, including more than 1,000 anesthetists, have sat for the NLE to date.

Using NLEs with questionable validity may allow poorly qualified graduates to join the practicing workforce and provide unsafe healthcare (false positives). On the other hand, a less valid examination may unjustly prevent otherwise competent graduates from joining the workforce (false negative), hindering the UHC aspirations [24, 25]. However, studies examining whether better performance in NLEs is associated with better quality of clinical care are few and limited to high-income countries. The available few studies reported a predictive relationship between medical doctors’ licensing examination scores and measures of standards of practice [26], quality of primary care [27], patient outcome after specific disease conditions [28], and medicolegal complaints reported to regulators [29]. However, to our knowledge, no study has assessed the association between NLE scores and quality of care (QoC) provided by associate clinicians in the context of low-income countries.

This study aimed to assess the association between Ethiopian anesthetist NLE score and the quality of perioperative care. We hypothesized that surgical patients who received anesthetic care from anesthetists with higher NLE scores would have a better quality of care measured in terms of standards for safe anesthesia practice, critical incidents, and satisfaction.

## Methods

### Study design

A longitudinal observational study design was employed to examine the relationship between NLE scores and three selected QoC indicators. The standards for safe anesthesia practice, critical incidents, and patient satisfaction were used to measure the QoC.

### Study setting

The study was conducted in public hospitals in the Amhara region, northwestern part of Ethiopia. There were 82 operational public hospitals in the region providing surgical care during the study period [30]. We randomly selected 18 study hospitals from the three-tier health system (nine primary, six general, and three referral) from nine of the thirteen zones of the region that were safe for travel during the study period.

### Study population

We studied surgical patients who received anesthetic care from the target group of anesthetists (*n* = 56) who completed their baccalaureate anesthesia training after 2019, passed the NLE, and were practicing anesthesia at any of the study site hospitals. To address study objectives, we reviewed medical records and conducted patient satisfaction surveys.

### Sampling and sampling procedure

All eligible patients who had undergone surgery during the one-month data collection period (January 8 to February 7, 2023) had their medical records reviewed longitudinally to determine the standards for safe anesthesia practice and perioperative critical incidents. Thus, we did not have a predefined sample size for medical record reviews. The medical records were thoroughly reviewed at least three times: before surgery, immediately after surgery, and once the patients were transferred to their destination wards. Data collectors were advised to go through multiple rounds of record reviews to ensure data accuracy and completeness. On the other hand, the sample size for the patient satisfaction survey was calculated using a single population proportion formula based on a previous Ethiopian study report of a 74% satisfaction rate, a 95% confidence interval, a 5% margin of error, and a 35% nonresponse rate [31]. The total sample size for the satisfaction survey was 400. For the satisfaction survey, we employed systematic sampling by enrolling every eligible patient in any of the primary hospitals, every third patient in any of the general hospitals, and every second patient in any of the tertiary hospitals. This resulted in 99 satisfaction surveys being collected weekly (three from each primary, six from each general, and 12 from each tertiary hospital). Weekly recruitments for satisfaction surveys were stopped at each hospital when the target number was reached. The remaining four surveys were collected from four general hospitals on the final two days of the study month.

### Inclusion and exclusion criteria

Adult patients undergoing surgery under general, regional, or sedation anesthesia and receiving care from target group anesthetists were eligible for medical record review. From this, those who had had uncomplicated surgery and were in a clinically stable condition after surgery were eligible for the patient satisfaction survey. The exclusion criteria were patients under the age of 18, those who were critically ill, and patients undergoing high-risk surgeries. The first-attempt NLE scores of the targeted anesthetists were obtained from the Ministry of Health. Other anesthetist-related study data were collected from the respective clinical anesthesia department records and the hospital human resource offices.

### Data collection tools

A 58-item checklist was used to collect standards for safe anesthesia practice and critical incident data from medical records (supplementary file 1). The tool was divided into five sections: patient sociodemographic data, preoperative status and care, intraoperative care and outcome, postoperative care and outcome, and anesthetist-related data.

First, ten items targeted at measuring the highly recommended *standards for safe anesthesia practice* (as met or not met) as identified by the WHO-World Federation of Societies of Anesthesiologists (WFSA) [32] and the Ethiopian government [33]. These Standards are intended to guide anesthetists, facilities, and governments in maintaining and enhancing the quality of anesthesia services. The term “highly recommended” relates to obligatory standards.

Second, twenty-six items aimed at assessing the occurrences of important *critical perioperative incidents* (as yes or no) listed by the Royal College of Anesthetists [34] and an African RCT study [35]. A critical incident is defined as any preventable mishap related to the planning or provision of anesthesia care that results in or could have resulted in an unfavorable patient outcome. The critical incidents assessed include death involving anesthesia, failed tracheal intubation and mask ventilation, dental trauma, aspiration pneumonitis, renal insufficiency, cardiac arrhythmias (brady or tachyarrhythmias), severe hypo or hypertension (a 30% or more change in blood pressure from baseline), severe hypothermia (core body temperature less than 36 °C), cerebrovascular accident, failed regional anesthesia, anaphylaxis, pain, postoperative nausea and vomiting, and surgery cancellation for anesthesia reasons. The remaining 22 items were designed to assess anesthetist characteristics and other important confounding variables, including anesthetists’ engagements relevant in-service training (IST).

Third, we applied a 15-item modified version of the Consumer Assessment of Healthcare Providers and Systems (CAHPS) Surgical Care Survey [36] with a five-point Likert scale to assess *patient perception and satisfaction* with anesthesia care (supplementary file 2). The satisfaction survey was translated into the local language, Amharic, and piloted with four volunteers before the actual data collection.

### Data collection procedures

Eighteen senior anesthetists practicing in the respective study hospitals (but not the target group anesthetists) collected study data between January 8 and February 7, 2023, by reviewing medical records and conducting patient satisfaction surveys.

After a two-day intensive data collection training with mock sessions, the data collectors first met with the respective hospital directors or CEOs, asking them to approve data collection. The data collectors then met with ward and operating room heads to secure permission to access medical records. Standards for safe anesthesia practice and critical incident data were longitudinally gathered from patient medical records, operation room schedules, and departmental weekly critical incident audit reports using a Kobo Toolbox application on organization-owned tablets. Other anesthetist-related study data were collected using the same digital platform from anesthesia department records and hospital HR offices.

For the patient satisfaction surveys, the trained data collectors first identified eligible candidates from the roster. They then met with each eligible patient in their respective wards postoperatively to unveil the study objectives and obtain informed verbal consent to complete the satisfaction survey. Within 4–8 h of their transfers from post-anesthesia care units, the trained data collectors interviewed consented patients and completed the satisfaction survey in their destination wards. Data collectors assessed any discomfort or pain and informed patients to report if any, though no complaints were reported. When the weekly target surveys were completed, the data collectors sealed and stored the completed surveys until the principal investigator/ supervisors collected them biweekly. Completed surveys were stored and locked in a department cabinet with locks accessible to the data collectors only. Three trained supervisors oversaw the entire data collection procedure and collected the sealed completed satisfaction surveys every other week.

After obtaining the list of target anesthetists from the study hospitals, the primary investigator (YMA) gathered their national licensing examination scores from the Ministry of Health (MOH) maintaining ethical considerations.

### Data analysis

NLE scores were first standardized using z-scores to ensure comparability across the cohorts of examinees. We employed descriptive statistics to summarize key study variables related to anesthetists and patients. The relationship among the three quality measures was investigated using Pearson’s correlation analysis and interpreted using recommended ranges to determine the strength and direction of associations [37].

We run three separate multivariable regression analyses to determine whether the NLE score (as measured by the percentage of correctly answered items) predicted the three QoC indicators. A p-value of less than 0.25 on bivariate regression was considered to select variables for the multivariable model. Multivariable linear regression was used to assess the relationship between the NLE score (an independent variable) and the continuous mean percentage standards for safe anesthesia practice achieved (a dependent variable). The mean percentage standards for safe anesthesia practice was calculated by averaging the percentage of anesthesia practice standards met in each patient’s care. Similarly, multivariable linear regression was employed to investigate the relationship between NLE scores and patient satisfaction. For this analysis, the mean percentage satisfaction score was computed by averaging individual patient satisfaction scores (total Likert score divided by maximum expected score multiplied by 100%). Besides, the relationship between NLE scores and critical incidents was investigated using multivariable logistic regression. For this analysis, the dependent variable (occurrence of a critical incident) was dichotomized by classifying patients with at least one critical incident as ‘yes’ and those with none as ‘no’.

To minimize the effect of confounding variables, key variables that may increase the likelihood of critical perioperative incidents were proactively identified from the literature and disaggregated data collected. The variables identified from the literature as possible confounders were related to the patients’ underlying medical conditions, the type of anesthesia administered, and the surgery performed. By combining risk scores for patient co-morbidity (i.e., the American Society of Anesthesiologists [ASA] physical status classification system) [38] and the complexity of surgery (i.e., the Silverman-Holt Aggregate Preoperative Evaluation [SHAPE] surgical score) [39], we stratified and adjusted the analysis for composite patient risk to arrive at a composite patient risk score. This composite patient risk score was computed by adding the ASA and SHAPE scores for each patient.

The linear regression analysis outputs are presented as an unstandardized B-coefficient and 95% confidence interval (CI), while Odds Ratio (OR) and 95% CI are used to present the logistic regression outputs. A p-value of less than 0.05 was considered statistically significant for all tests unless otherwise specified. All analyses were performed using SPSS ver27 (IBM Corp.)

## Result

### Characteristics of anesthetists

A total of 56 anesthetists were assessed in this study. The majority of anesthetists were males (92.9%). The average years of work experience was 2.1 years (SD = 1.1). The overall mean first-attempt NLE score was 58.8% (SD = 8.5), while the pass rate was 98.2% (table not shown).

### Number of patients by the attending anesthetist characteristics

The target group of anesthetists managed a total of 991 surgical patients during the study period. The majority of patients were managed by male anesthetists (92.5%) who passed the NLE on the first attempt (98.6%) and anesthetists who reported not receiving any in-service training within 12 months of data collection (85.7%). The median number of patients managed by each anesthetist over the month was 24 (Table 1).

**Table 1:** Number of surgical patients by anesthetist-related variables, January 8 - February 7, 2023, *n* = 991

Variable		# of patients	%
Gender of anesthetist	Male (*n* = 52)	843	92.5
	Female (*n* = 04)	68	7.5
Status on first NLE attempt	Pass (*n* = 54)	898	98.6
	Fail (*n* = 02)	13	1.4
Year of anesthetist practice since completing university training	< 1 year (*n* = 13)	205	22.5
	1–2 years (*n* = 22)	337	37.0
	Above 2 years (*n* = 21)	369	40.5
Any relevant IST to anesthetist in the past 12 months	Yes (*n* = 14)	130	14.3
	No (*n* = 42)	781	85.7
Number of patients managed by anesthetists (Median [IQR])		24 [14]	

Abbreviations. NLE, national licensing examination; IQR, inter-quartile range; IST, in-service training

### Patient characteristics by clinical status, type of care, facility, and quality of care indicators

Nearly all patients (97.6%) were medically stable (ASA 1 or 2), and the majority (63.7%) presented as an emergency and underwent surgery under spinal anesthesia (61.1%), with the frequent surgical category being gynecology and obstetrics (54.3%). The mean percentage standards for safe anesthesia practice achieved was 69.1% while the mean percentage satisfaction score was 85.22%. Three of the five patients developed at least one critical incident perioperatively (Table 2). A total of 1120 critical incidents on the 911 patients were documented, with the top three most common incidents being severe intraoperative hypo/hypertension (30.4%), severe hypothermia (24.3%), and cardiac arrhythmias (15.9%). There were also three deaths (table not shown).

**Table 2:** surgical patient characteristics by demographic, hospital, surgical workforce, and patient outcome characteristics, January 8 - February 7, 2023

Variable	Characteristics	*N*	%
*Patient characteristics*			
Gender (*n* = 911)	Male	277	30.4
	Female	634	69.6
Type of surgery (urgency) (*n* = 911)	Elective	331	36.3
	Emergency	580	63.7
Surgical category (*n* = 911)	Gynecology and obstetric	495	54.3
	Bowel, hepato-biliary, ENT, and vascular	223	24.5
	Plastics and breast	63	6.9
	Urology	42	4.6
	Neurosurgery	5	0.6
	Other surgeries	83	9.1
American Society of Anesthesiologists [ASA] grading (*n* = 911)	Grade 1 (score = 1)	277	30.4
	Grade 2 (score = 2)	612	67.2
	Grade 3 (score = 3)	21	2.3
	Grade 4 (score = 4)	1	0.1
[Silverman-Holt Aggregate Preoperative Evaluation [SHAPE] surgical score (*n* = 911)	Minor (score = 1)	83	9.1
	Low intermediate (score = 2)	187	20.5
	Intermediate (score = 3)	636	69.8
	High intermediate (score = 4)	5	0.6
Composite perioperative patient risk score (ASA **+** SHAPE scores) (Mean [SD])		4.34 [0.96]	
*Anesthetic care and facility characteristics*			
Type of anesthesia (*n* = 882)*	General anesthesia	298	32.7
	Spinal anesthesia	557	61.1
	Sedation, PNB, and others	27	3.0
Qualification of the surgeon (*n* = 881)*	Physician specialist	492	54.0
	Associate clinician (IESO)	389	42.7
Level of hospital (*n* = 911)	Primary	172	18.9
	General	444	48.7
	Tertiary	295	32.4
*Quality of care (QoC) indicators*			
Perioperative critical incidents (*n* = 911)	No critical incident	343	37.7
	One or more critical incidents	568	62.3
Standards for safe anesthesia practice (*n* = 911)	Percentage standards for safe anesthesia practice met (Mean percentage [SE, 95% CI for SE])	69.1 [0.60, 0.58–0.63]	
Patient satisfaction (*n* = 400)	Percentage patient satisfaction score (Mean percentage [SE, 95% CI for SE])	85.2 [0.5, 0.46–0.54]	

Abbreviations.: ENT, ear, nose and throat surgeries; SE, standard error of mean percentages; CI, confidence interval; PNB, peripheral nerve blocks; IESO, Integrated Emergency Surgical Officers. Note: *some missing data

### Correlations between the three quality of care indicators

The standards for safe anesthesia practice and patient satisfaction were positively correlated (*r* = 0.12; 95% CI = 0.02–0.22; *p* = 0.014). On the other hand, the standards for safe anesthesia practice was inversely correlated with critical incidents (*r* = -0.12; 95% CI -0.22, -0.02; *p* = 0.016), as did critical incident and patient satisfaction (*r* = -0.15; 95% CI -0.23, -0.05; *p* = 0.002).

### Multivariable linear and logistic regressions

The anesthetist NLE score was able to predict all three QoC indicators before adjustment for covariates related to the patients’ underlying medical conditions, the type of anesthesia administered and surgery performed, experience and training of anesthetist, and facility type. However, after controlling for patient, anesthetist, facility, and clinical care essential confounding variables, the NLE score was only able to predict the critical incidents: the odds of developing one or more critical incidents declined by 18% for every 1% point increase in the total NLE score [OR = 0.82; 95% CI, 0.70, 0.96; *p* = 0.016] (Table 3). Similarly, adding each QoC measure to one another’s regression model (as applied) did not yield significant results.

**Table 3:** Multivariable linear and logistic regressions on predictor relationship between NLE score and QoC indicators (standards for safe anesthesia practice, perioperative critical incidents and patient satisfaction); January 8 - February 7, 2023

Outcomes ◊	Standards for safe anesthesia practice (multivariable linear regression)		Perioperative incident (Multivariable logistic regression)		Patient satisfaction (Multivariable linear regression)	
Predictors ↓	Unstandardized B [95% CI]	P value	OR [95% CI]	P value	Unstandardized B [95% CI]	P value
Anesthetist gender (ref: female)	-5.16 [-10.30, -0.02]	0.049	1.49 [0.76, 2.94]	0.241	-2.87 [-6.45, 0.70]	0.115
Total number of patients handled	-	-	0.99 [0.98, 1.01]	0.510	-0.08 [-0.17, 0.02]	0.125
Year of clinical practice since taking NLE	-1.04 [-2.13, 0.05]	0.061	-	-	1.36 [0.52, 2.21]	0.002*
Presence of another anesthetist for assistance (ref: no)	2.46 [0.07, 4.85]	0.044	0.53 [0.39, 0.72]	< 0.001*	1.68 [-0.20, 3.56]	0.080
Any relevant IST to anesthetist in the past 12 months (ref: no)	8.73 [4.53, 12.92]	< 0.001*	0.35 [0.21, 0.61]	< 0.001*	4.81 [1.56, 8.07]	0.004*
Qualification of the surgeon (ref: associate clinicians)	-3.75 [-6.31, -1.19]	0.004*	0.86 [0.58, 1.28]	0.451	1.88 [-0.48, 4.25]	0.119
Composite patient risk score	-0.94 [-2.23, 0.36]	0.155	1.34 [1.13, 1.59]	0.001*	1.45 [0.39, 2.51]	0.008*
Hospital level (ref: tertiary hospital):						
Primary Hospital	-	-	0.42 [0.23, 0.77]	0.005*	-1.00 [-4.38, 2.38]	0.560
General Hospital	-	-	1.02 [0.66, 1.57]	0.934	-4.59 [-7.24, -1.94]	0.001*
Type of anesthesia (ref: general anesthesia with intubation)						
Sedation and peripheral nerve blocks	-	-	0.45 [0.19, 1.07]	0.071	-	-
Spinal anesthesia	-	-	-	-	0.16 [-1.81, 2.13]	0.876
First-attempt total NLE score	-0.70 [-1.97, 0.57]	0.279	0.82 [0.70, 0.96]	0.016*	0.40 [-0.59, 1.38]	0.427

Note: empty cells indicate non-significant findings on bivariate regression (*p* > 0.25) and thus not included in multivariable regression. * p statistically significant after adjustment for multiple tests using Bonferroni correction

On the other hand, it was notable that participation in relevant in-service training (IST) was a consistently strong statistically significant predictor of all three QoC measures. Patients have 2.86 times the odds of sustaining one or more critical incidents when managed by anesthetists who did not receive IST than those who did (95% CI, 1.64, 4.76; *P* < 0.001; note reverse OR calculation here). Besides, patients who received care from anesthetists with at least one IST had 8.73% higher standards of care for safe anesthesia practice (95% CI = 4.53, 12.92; *P* < 0.001) and 4.81% higher satisfaction scores (95% CI, 1.56, 8.07; *P* = 0.004) (Table 3).

Finally, the sensitivity and specificity of NLE at a 50% cut-off score threshold revealed a relatively higher sensitivity but lower specificity across the three QoC measures. Accordingly, the sensitivity and specificity of the NLE at this threshold were as follows: 0.82 and 0.10 for predicting no critical incidents, 0.87 and 0.28 for meeting at least 50% of perioperative standards, and 0.84 and 0.03 for achieving at least 80% patient satisfaction score.

## Discussion

The most important finding of our study is that patients who receive anesthetic care from anesthetists with higher NLE scores have lower odds of developing one or more critical incidents when controlling for patient, clinical care, facility, and anesthetist characteristics. Similarly, having another assistant anesthetist present during intraoperative anesthetic care reduced the likelihood of critical incidents.

Our findings are similar to those seen in the United States, where cognitive licensing examination scores of international medical school graduates exhibited an inverse relationship with mortality [28]. However, the odds reduction we reported for each point increase in NLE score (18%) is larger than that in the cited study (0.2%). The likely explanation is that we set a low threshold for including other critical incidents in addition to mortality, whereas the other study only counted mortality. Our finding has important policy implications as it supports the theory that the Ethiopian anesthetist NLE, in its current form, might filter out anesthetists that may potentially cause harm to patients. Establishing a culture of confidential inquiries into serious critical incidents can help health facilities learn and prevent similar events from happening again [40]. Besides, the positive patient outcomes associated with an assistant’s presence can help stakeholders make informed decisions during facility staffing standard revisions.

On the other hand, it is intriguing to note that NLE scores do not predict standards for safe anesthesia practice and patient satisfaction. This is in contrast to other studies on medical doctors in the United States, which reported a statistically significant relationship between licensing examination score and QoC, as measured by record reviews of screening, consultation, and medication prescription practices [27], peer assessment of the quality of their care [26], and complaints reported to regulators [29]. This discrepancy may be attributed to variations in the QoC measurement approaches and the incompleteness of the medical records we reviewed. Studies revealed substandard perioperative documentation practices among Ethiopian anesthetists, with up to 20% of preoperative, 10% of intraoperative, and 30–50% of postoperative recordings being incomplete [41, 42].

Our finding on the inverse predictive relationship between anesthetist NLE score and perioperative critical incidents calls for strengthening the Ethiopian NLE to establish a patient outcome-based pass score. However, the lack of relationships between the NLE score and standards for safe anesthesia practice and patient satisfaction commends establishing a system for verifying that graduates are competent in soft, non-technical skills that are not assessed in this cognitive assessment [43]. Besides, the failure to meet nearly one-third of the mandatory standards (mean percentage standards for safe anesthesia practice met = 69.14%) takes us beyond the NLE and into the teaching institutions. Anesthesia teaching institutions should improve learning and assessment practices for non-technical skills that require deliberately designed contemporary interventions, including workplace-based assessments [44, 45]. The Amhara Regional Health Bureau and study site hospitals can also benefit from cultivating a culture of institutional clinical audit to ensure compliance with care delivery standards [46]. Meanwhile, the MOH shall consider tightening the NLE cutoff pass scores by estimating NLE score thresholds based on positive patient outcomes, decreasing false positives, and enhancing the exam’s specificity.

On the other hand, an intriguing result from our study was the statistically significant association between participation in recent (within a year) in-service training and all three QoC indicators. Other studies reported congruent findings where engagement in continuing professional development (CPD) activities improved professionals’ behavior and patient outcomes [47, 48]. Our finding has particular policy implications for the newly mandated regulatory practice of CPD activities for re-licensure [49]. Our results favor implementing targeted and cost-effective CPD activities in areas of frequent practice and recurring critical incidents. To get the most out of it, regulators must strengthen the system for monitoring CPD compliance, including ensuring the yearly minimum credits and linking it to license renewal (note that only a quarter of anesthetists in our study took at least one training). Aside from maintaining competence, CPD opportunities can help to motivate and retain anesthetists [50].

### Strength and limitations

Our study has several strengths. First, to the best of our knowledge, this is the first study to examine the relationship between licensing examination scores of associate clinicians and the quality of patient care they provide in a low-income country. Second, multiple health facilities and surgical cases were involved, giving a representative sample. Third, because we had a relatively large sample size, multiple confounding variables were identified proactively, disaggregated data were gathered, and their effect was controlled. Fourth, we collected both primary (through surveys) and secondary (through medical records reviews) data, allowing us to assess the different QoC dimensions. Finally, we adopted and employed validated assessment tools facilitating comparisons.

However, our study also has important limitations. We relied on medical record review to determine critical incidents and standard care practices. Our results might have under- or overestimated findings due to incomplete records for which we took all eligible cases to compensate for losses.

## Conclusion

The current study will add to the body of validity evidence for the Ethiopian anesthetist NLE. The NLE score showed an inverse relationship with critical perioperative incidents, supporting its use in its current form. However, the lack of a relationship between NLE scores and the other two QoC indicators warrants further scrutiny. This upholds the need for establishing a system to verify soft skills using a sequential OSCE as part of the NLE or considering a more robust portfolio of on-the-job performance assessments as a requirement to sit for NLEs. Future research should look into methods for establishing QoC-based NLE cut-off scores and developing more refined quality measures. Longitudinal follow-up studies on specific bellwether surgical procedures should be conducted to determine whether the exam predicts the primary roles of these graduates.

### Electronic supplementary material

Below is the link to the electronic supplementary material.

**Supplementary file 1**: Quality of perioperative patient care survey

**Supplementary file 2**: Patient satisfaction survey (English version).

## Data Availability

The datasets generated and analyzed during the current study are not publicly available due to privacy and ethical concerns. However, they are available from the corresponding author upon reasonable request.

## Abbreviations

LMICs: Low- and Middle-Income Countries
NLE: National Licensing Examination
QoC: Quality of Care
