# Supplementary material for: Does higher performance in a national licensing examination predict better quality of care? A longitudinal observational study of Ethiopian anesthetists
Source: BMC Anesthesiol. 2024 May 27;24:188. doi: 10.1186/s12871-024-02575-w (PMC11129401; doi:10.1186/s12871-024-02575-w)
Supplement: Supplementary file 1 — Quality of perioperative patient care survey [file 12871_2024_2575_MOESM1_ESM.pdf]

Quality of perioperative patient care survey

General patient information

(Data source: patient medical records)

Age: ☐☐☐ years Sex: ☐ Male ☐ Female

ASA: ☐ I ☐ II ☐ III ☐ IV ☐ IV ☐ NR Mallampati: ☐ I ☐ II ☐ III ☐ IV ☐ NR

Comorbidity *(tick all that apply)*: ☐ Hypertension ☐ COPD/ Asthma ☐ DM ☐ HIV/AIDS

Category of surgical procedure *(tick the single most appropriate)*:

☐ Obstetrics ☐ Gynecology ☐ Trauma and Orthopedic ☐ Thoracic ☐ Urology ☐ Ophthalmic

☐ GI & hepatobiliary ☐ Neurosurgery ☐ ENT & maxillofacial ☐ Thyroid/ breast ☐ Other

Indication for surgery: ☐ Cesarean delivery ☐ Trauma ☐ Infection ☐ Non-communicable

Type of surgery (urgency): ☐ Elective ☐ Emergency

Risk of surgery: ☐ Minor ☐ Intermediate ☐ Major

Level of hospital: ☐ Primary ☐ General ☐ Referral

Preoperative information

(Data source: patient medical records)

A comprehensive pre-anesthetic patient assessment performed by the anesthetist ☐ Yes ☐ No

Preoperative patient preparation is done by the anesthetist ☐ Yes ☐ Partially ☐ No

Informed consent secured (a day before surgery for electives) ☐ Yes ☐ No

A comprehensive perioperative pain management plan developed ☐ Yes ☐ No

Postoperative nausea and vomiting management plan developed ☐ Yes ☐ No

Perioperative management plan for patients' current medications developed ☐ Yes ☐ No ☐ NA

Prophylactic antibiotics administered within 1h before incision ☐ Yes ☐ No

Intraoperative information

(Data source: patient medical records and weekly departmental morbidity and mortality reports)

Surgery canceled on the day of surgery for anesthetic reasons other than an acute medical condition

☐ Yes ☐ No

Type of anesthesia administered *(tick all that apply)*: ☐ GA with intubation ☐ GA with LMA

☐ Sedation ☐ Spinal anesthesia ☐ Peripheral nerve block ☐ Other

Surgery canceled while receiving anesthetic care ☐ Yes ☐ No

Failed tracheal intubation ☐ Yes ☐ No ☐ NA (for regional)

Difficult ventilation with a mask ☐ Yes ☐ No ☐ NA (for regional)

Accidental dental trauma during airway management ☐ Yes ☐ No ☐ NA (for regional)

Gastric content aspirated while receiving anesthetic care ☐ Yes ☐ No ☐ NA (for regional)

Failed regional anesthesia (e.g. failed spinal anesthesia) ☐ Yes ☐ No ☐ NA (for Gas and PNB)

Medication error ☐ Yes ☐ No

Transfusion ☐ Yes, one unit ☐ Yes, more than one unit ☐ No

Anaphylaxis of any cause and drug reactions ☐ Yes ☐ No

Severe intraoperative hypo/hypertension ☐ Yes, once ☐ Yes, more than once ☐ No

Patient body temperature was 36°C or below ☐ Yes, once ☐ Yes, more than once ☐ No

Cardiac arrhythmias ☐ Yes, once ☐ Yes, more than once ☐ No

Decision to baby out time (for cesarean delivery)? ☐☐ Minutes

APGAR score of the newborn 1st minute ☐☐ 5th minute ☐☐

Total number of anesthetic and adjuvants drugs used by the anesthetist: ☐☐

WHO surgical safety checklist used, filled, and documented ☐ Yes ☐ No

Complete intraoperative patient records produced and maintained ☐ Yes ☐ No

Cardiac arrest during or within 48 hours of anesthetic care ☐ Yes ☐ No

Another anesthetist was present to assist the responsible anesthetist ☐ Yes ☐ No

The responsible anesthetist receive technical update training in the past year? ☐ Yes ☐ No

Postoperative information

(Data source: patient medical records and weekly departmental morbidity and mortality reports)

Unplanned patient stay in the PACU or RR longer than 2 hours? ☐ Yes ☐ No

Problem with the patient airway in the PACU/ RR ☐ Yes ☐ No

A need for reintubation during or within 48 hours of anesthetic care ☐ Yes ☐ No

Respiratory arrest during or within 48 h of anesthetic care ☐ Yes ☐ No

Severe postoperative hypo/hypertension requiring intervention? ☐ Yes ☐ No

Renal insufficiency developing during or within 48 hours of anesthetic care ☐ Yes ☐ No

Cerebrovascular accident during or within 48 hours of anesthetic care ☐ Yes ☐ No

Peripheral nerve deficit during or within 48 h of anesthetic care ☐ Yes ☐ No

Post-dural puncture headache after spinal anesthesia ☐ Yes ☐ No

Complete patient handover notes documented with postoperative orders ☐ Yes ☐ No

Severe nausea and vomiting within 48 hours of procedure that require intervention ☐ Yes ☐ No

Patient body temperature was 36°C or below in PACU/ RR ☐ Yes ☐ No

Unplanned overnight admission for anesthetic reasons (for daycare surgeries)? ☐ Yes ☐ No

Anesthetist-related information

(Data source: MOH licensing exam database. Note: the PI will collect this data once and it will not be accessible to data collectors or anyone else except the codes assigned to each anesthetist)

Gender of the responsible anesthetist: ☐ Male ☐ Female Age: ☐☐ years

Joined university as (entry behavior): ☐ Direct entry (from high school) ☐ Post-basic (nursing)

Generation of training institution/ university: ☐ First ☐ Second ☐ Third ☐ Fourth

Year of graduation: ☐☐☐☐

Passed the licensing examination with a score of ☐☐%

Total number of attempts on the licensing exam: ☐☐
