# Supplementary material for: Does higher performance in a national licensing examination predict better quality of care? A longitudinal observational study of Ethiopian anesthetists
Source: BMC Anesthesiol. 2024 May 27;24:188. doi: 10.1186/s12871-024-02575-w (PMC11129401; doi:10.1186/s12871-024-02575-w)
Supplement: Supplementary file 2 — Patient satisfaction survey (English version). [file 12871_2024_2575_MOESM2_ESM.pdf]

English version of patient satisfaction survey

Code of managing anesthetist: _______

Dear madam/ sir,

I have indicated to you that I am supporting the Ministry of Health to conduct a research project on the impacts of introducing the anesthetist national licensing examination.

This questionnaire aims to gather data on your satisfaction and experience with the anesthesia service.

This survey will take about 30 minutes to complete.

You have already listened to my presentation during the consent.

PERIOPERATIVE PATIENT SATISFACTION SURVEY RATING

Please indicate your agreement or disagreement with the following statements about your experience during your surgical care.

S.Nº	Statement	Strongly Agree	Agree	Neither	Disagree	Strongly Disagree
QA_1.	During the visit with the anesthetist before the surgery, the anesthetist explained to me how I would feel after anesthesia	5	4	3	2	1
QA_2.	The anesthetist encourage me to ask questions	5	4	3	2	1
QA_3.	I was able to ask the questions I wanted	5	4	3	2	1
QA_4.	The information given to me by the anesthetist was understandable	5	4	3	2	1
QA_5.	I am satisfied with the amount of information given to me by the anesthetist	5	4	3	2	1
QA_6.	Talking with the anesthetist during the pre-operative visit make me feel calmer and more relaxed	5	4	3	2	1
QA_7.	The anesthetist considers my privacy in the operation and recovery area	5	4	3	2	1

S.№	Statement	Strongly Agree	Agree	Neither	Disagree	Strongly Disagree
QA _8.	I am satisfied with the treatment of nausea and vomiting after the surgery	5	4	3	2	1
QA_9.	I am satisfied with the pain therapy after the surgery	5	4	3	2	1
QA_10.	I found the anesthetist very professional	5	4	3	2	1
QA_11.	I have confidence in my anesthetist	5	4	3	2	1
QA_12.	I would recommend the anesthetist to others in my family	5	4	3	2	1
QA_13.	The overall quality of anesthetic care by the anesthetist is satisfying	5	4	3	2	1
QA_14.	I would want to have the same anesthetic care again	5	4	3	2	1
QA_15.	Based on this experience, I have a good understanding of the role the anesthetist played in my surgery	5	4	3	2	1
QA_16.	Using any number from 0 to 10, where 0 is the no pain and 10 is the worst pain, what number would you used to rate the pain you have (at 24hr postoperatively)?					
QA_17.	Using any number from 0 to 10, where 0 is the worst anesthetist possible and 10 is the best anesthetist possible, what number would you used to rate this anesthetist?					

Adopted from CAHPS and ASA satisfaction with anesthesia services questionnaire
